# Neonicotinoid Insecticides Alter the Transcriptome of Soybean and Decrease Plant Resistance

**DOI:** 10.3390/ijms20030783

**Published:** 2019-02-12

**Authors:** Jason A. Wulff, Mahnaz Kiani, Karly Regan, Micky D. Eubanks, Adrianna Szczepaniec

**Affiliations:** 1 Department of Entomology, Texas A&M University, College Station, TX 77843, USA; jsnwlff@gmail.com; 2 Department of Entomology, Texas A&M AgriLife Research, Amarillo, TX 79106, USA; Mahnaz.Kianifariz@ag.tamu.edu; 3 Department of Entomology, Penn State University, University Park, PA 16801, USA; kjr5470@psu.edu

**Keywords:** thiamethoxam, imidacloprid, spider mites, *Glycine max*, *Tetranychus cinnabarinus*

## Abstract

Neonicotinoids are widely used systemic insecticides that have been associated with spider mite outbreaks on diverse plants. These insecticides have complex effects on plant physiology, which have been speculated to drive enhanced performance of spider mites. We used RNA-Seq to explore how neonicotinoids modify gene expression in soybean thereby lowering plant resistance. We exposed soybean (*Glycine max* L.) to two neonicotinoid insecticides, thiamethoxam applied to seeds and imidacloprid applied as a soil drench, and we exposed a subset of these plants to spider mites (*Tetranychus cinnabarinus*). Applications of both insecticides downregulated genes involved in plant—pathogen interactions, phytohormone pathways, phenylpropanoid pathway, and cell wall biosynthesis. These effects were especially pronounced in plants exposed to thiamethoxam. Introduction of spider mites restored induction of genes in these pathways in plants treated with imidacloprid, while expression of genes involved in phenylpropanoid synthesis, in particular, remained downregulated in thiamethoxam-treated plants. Our outcomes indicate that both insecticides suppress genes in pathways relevant to plant–arthropod interactions, and suppression of genes involved in cell wall synthesis may explain lower plant resistance to spider mites, cell-content feeders. These effects appear to be particularly significant when plants are exposed to neonicotinoids applied to soybean seeds.

## 1. Introduction

Insecticides indirectly affect crop yield by eliminating herbivores that compromise plant productivity [1,2]. Until recently, it was assumed that insecticides did not directly affect plants. Recent research, however, suggests that a group of insecticides commonly used in agricultural crop protection, neonicotinoids, alter primary and secondary plant functions and reduce the ability of plants to defend themselves against invertebrate herbivores unaffected by the toxicity of these insecticides [3,4,5]. Given the widespread, almost ubiquitous use of neonicotinoids in agricultural crops and ornamental plants [6], exploring the consequences of these insecticides on plant transcriptome is a key step to understanding their broader impacts on plant physiology and plant–arthropod interactions. 

Neonicotinoids have complex, variable, and potentially beneficial effects on plant growth, vigor, and stress response. For example, research in multiple plant species has demonstrated an association between neonicotinoid applications and increased seed germination in bean (*Phaseolus vulgaris*) [7], root growth in wheat (*Triticum aestivum*) [8], and seedling vigor in the presence of weed competition in corn (*Zea mays*) [9]. Neonicotinoids can also alter stress tolerance of plants, including increased drought tolerance in soybean [10] and cold tolerance in wheat [11]. While the genetic mechanisms that underlie these benefits are often unknown, these insecticides induced expression of the salicylic acid pathway, which increased disease resistance in *Arabidopsis thaliana* [3]. However, these effects are not consistent, which has been exemplified in several studies that report little benefit from neonicotinoid exposure on germination or plant productivity in systems, such as soybean (*Glycine max*) [12,13]. 

Direct effects of neonicotinoids on plant physiology have been implicated in shaping plant–herbivore interactions. In particular, outbreaks of several genera of spider mites (Acari: Tetranychidae), which are cell-content feeding herbivores not susceptible to neonicotinoid insecticides [14], have been associated with applications of these insecticides in diverse plant systems including trees [15,16,17], shrubs [18,19], and crop plants [4,20,21]. These outbreaks can be severe in their magnitude. For example, numbers of twospotted spider mites (*Tetranychus urticae*) feeding on corn, cotton (*Gossypium hirsutum*), and tomato (*Solanum lycopersicum*) treated with neonicotinoid insecticides increased by 30%, 60%, and 200%, respectively [2]. A similar outcome was reported in field experiments in cotton, where neonicotinoid insecticide exposure resulted in spider mite densities that were two to three times greater than in the untreated plants [21]. Toxicity of neonicotinoid-treated plants to spider mite predators likely contributed to the outbreaks, but elimination of natural enemies alone did not explain high populations of these pests in field experiments [15]. Instead, outbreaks of spider mites were linked to suppression of host plant defense against these herbivores. This was documented in cotton and corn plants that were exposed to thiamethoxam and clothianidin seed treatments, respectively, and exhibited suppressed transcription of several genes involved in plant resistance and lowered concentrations of plant hormones involved in plant defense against arthropods [4].

Interactions between changes in plants triggered by neonicotinoids and defenses induced by spider mite herbivory are likely to be complex. Phytohormone and gene expression analysis in multiple plant species indicate that a defense pathway governed by the plant hormone jasmonic acid (JA) is the predominant inducible plant defense employed against spider mite feeding [22,23,24,25,26], albeit a plant hormone, salicylic acid (SA) involved primarily in pathogen defenses can be induced by spider mite herbivory as well [27]. In fact, strong induction of the SA pathway was previously implicated in increased population growth of spider mites by suppressing induction of the JA pathway via the antagonistic cross-linkage (“cross-talk”) between these plant defensive pathways [22,28,29]. However, there is significant variation in incidence and strength of the JA–SA cross-talk across plants [30] and spider mites elicit responses regulated by both phytohormones [27,31]. It is noteworthy that the neonicotinoids can affect the SA pathway [3,4], which could potentially interfere with the JA-regulated defense response against spider mites. A previous study demonstrated a reduction in concentrations of 12-oxophytodienoic acid (OPDA), a precursor of JA in cotton (*Gossypium hirsutum*) and corn (*Zea mays*) grown from seeds treated with neonicotinoid insecticides [4]. Notably, alteration of other pathways can affect plant physiology and impact plant–arthropod interactions. For instance, repression of the cell wall and lignin genes are implicated in increased susceptibility to herbivory [32,33,34], and the neonicotinoid insecticide thiamethoxam has been previously shown to affect genes regulating cell wall biosynthesis in soybean (*Glycine max*) [5].

The goal of this research was to quantify the global transcriptional responses of a crop plant, soybean, to two neonicotinoid insecticides: thiamethoxam applied as seed treatments and imidacloprid applied as a soil drench. Thiamethoxam seed treatments represent one of the most common methods of neonicotinoid applications in crop plants [6], and imidacloprid soil drenches are prevalent in vegetable [35,36,37,38] and fruit production [39]. We examined how the interaction between spider mite herbivory and neonicotinoid exposure changed the soybean transcriptome, and quantified the effects of neonicotinoid seed treatments on the abundance of spider mites (*Tetranychus cinnabarinus*) on soybean. We hypothesized that the neonicotinoids would alter the transcriptome of soybean and affect plant processes that impact plant resistance to spider mites. Outbreaks of these arthropods following exposure to neonicotinoid insecticides have been reported previously [2,13,14,15,16,17,18], but this study is the first to explore global transcriptome changes in plants exposed simultaneously to the neonicotinoids and the herbivore that may explain reduced plant resistance to spider mites. 

## 2. Results

### 2.1. Neonicotinoid Insecticides Alone and in Combination with Spider Mite Herbivory Altered the Transcriptome of Soybean Plants

Sequencing of 18 libraries generated 12.8 to 29.6 million reads from individual samples, and 8.08 to 18.97 million reads were uniquely mapped to a soybean reference genome (Gmax_275_Wm82.a2.v1). On average, 98% of the reads mapped to the exonic regions (Supplementary File 1). 

**Figure 1:**
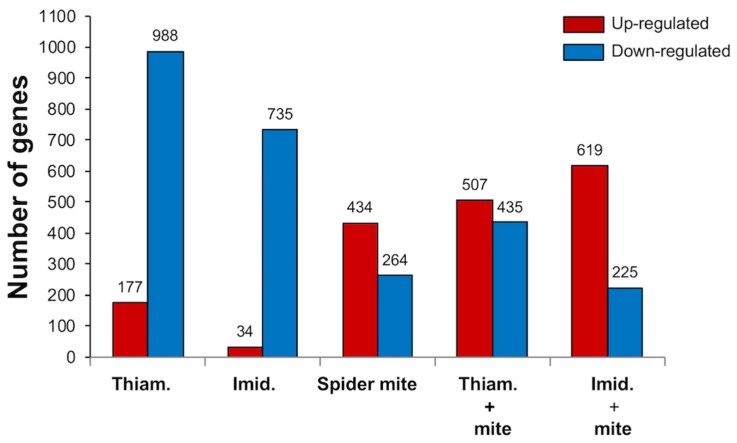
Differentially expressed genes (DEGs) in soybean plants exposed to thiamethoxam (Thiam.), imidacloprid (Imid.), spider mites, and the combination of the insecticides and spider mites. DEGs were defined as having a fold change ≥2 or ≤−2 with a false discovery rate (FDR) adjusted *p*-value < 0.05.

A total of 4418 differentially expressed genes (DEGs) were recovered, and 60% of them were down-regulated. Neonicotinoid insecticide exposure in the absence of spider mites suppressed the highest number of genes, with 988 and 735 genes down-regulated in response to thiamethoxam and imidacloprid, respectively (Figure 1). A relatively low number of genes were up-regulated in these plants, 177 for thiamethoxam- and 34 for imidacloprid-treated soybean. On the other hand, exposure to spider mite herbivory alone elicited a general induction of genes, with 434 transcripts up-regulated and 264 genes suppressed. Similarly, the majority of genes differentially expressed in plants exposed to neonicotinoids and spider mites were up-regulated, albeit soybean treated with thiamethoxam maintained a relatively high number of suppressed genes. These plants downregulated 435 transcripts and induced 507 genes. It is noteworthy that imidacloprid exposure and spider mite herbivory elicited the highest number of up-regulated DEGs, 619, while only 225 genes were suppressed. 

**Figure 2:**
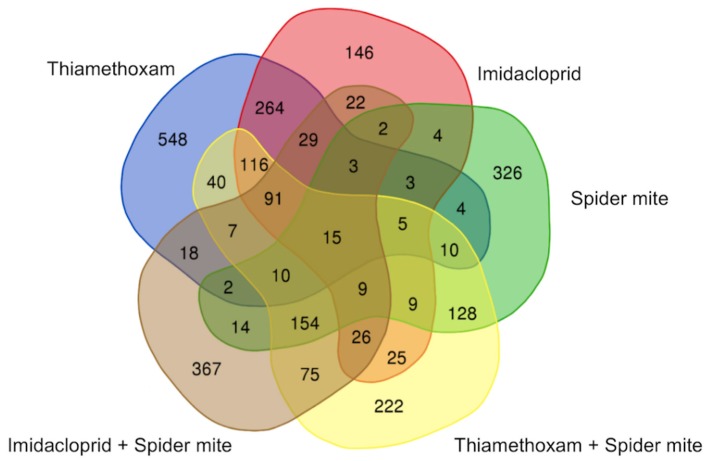
Venn diagram representing overlap in DEGs across treatments and DEGs unique to each of the treatments. DEGs were compared among soybean plants exposed to thiamethoxam, imidacloprid, spider mites, and the combination of either neonicotinoid insecticide and spider mites. All overlapped and unique DEGs are presented in the diagram.

The overlaps among DEGs representing changes in soybean plants across treatments were further illustrated in Venn diagram (Figure 2). Thiamethoxam-treated soybean had the highest number of uniquely expressed genes (548), while imidacloprid-treated plants had the fewest (146). Imidacloprid-treated and untreated soybean exposed to spider mites expressed a comparable number of unique transcripts—367 and 326, respectively, while 222 unique genes were expressed in plants exposed to thiamethoxam and spider mite herbivory. 

### 2.2. Neonicotinoid Insecticides and Spider Mites Affect Multiple Plant Pathways

**Figure 3:**
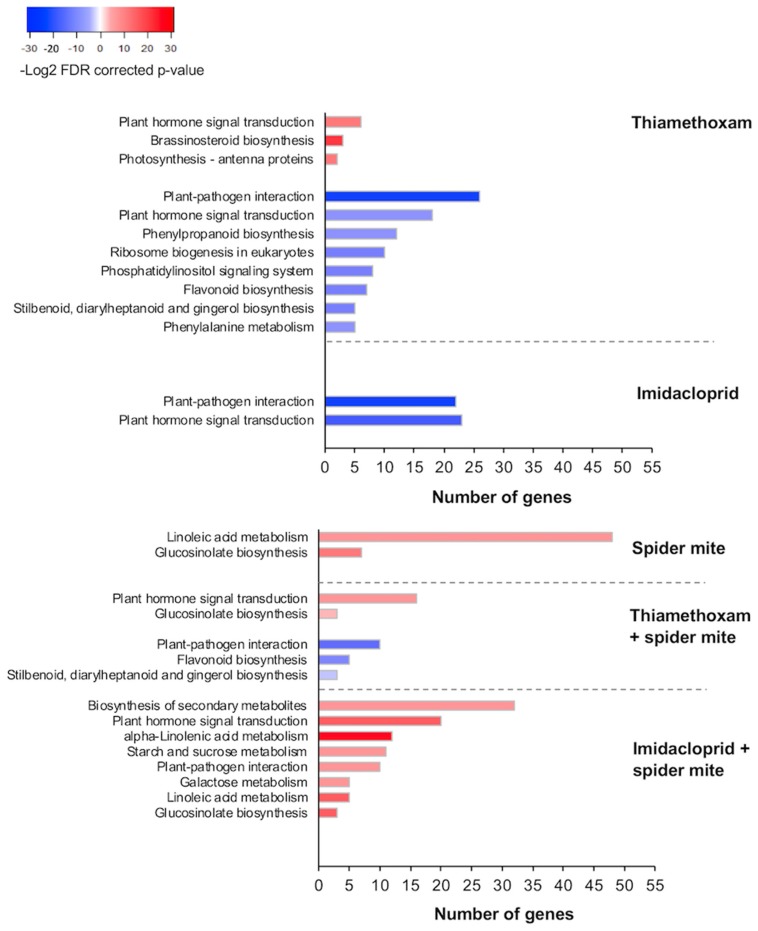
Pathways enriched in DEGs from soybean plants in response to thiamethoxam, imidacloprid, spider mite, and the combination of both insecticides and spider mites. Enriched pathways were selected based on an FDR corrected *p*-value < 0.05. Color of the bars refers to the −log2 corrected *p*-value of the respective enrichment pathway, red colored bars up-regulated genes, and blue colored bars indicate down-regulated genes. Length of the bar refers to the number of genes within the respective pathway, and the darker the color, the higher statistical significance.

Pathway enrichment analysis revealed that the insecticides altered a number of key plant pathways in the absence and presence of the mites (Figure 3). Thiamethoxam treatments alone affected the highest number of plant pathways, and the majority of these effects were suppressive. Transcriptome changes in plants treated with thiamethoxam included down-regulation of genes involved in plant hormone signal transduction, plant–pathogen interactions, and biosynthesis of several defensive compounds. Further, imidacloprid exposure alone had a strong and exclusively suppressive effect on plant–pathogen interaction and plant hormone signal transduction pathways. Spider mites alone and in combination with either neonicotinoid insecticides generally induced expression of genes in a number of plant pathways involved in primary and secondary metabolism. The only exception to this trend was thiamethoxam-treated plants, where spider mite herbivory had the weakest effect on induction of the plant hormone transduction and glucosinolate biosynthesis and simultaneously downregulated several pathways, including the plant–pathogen interactions pathway. It is noteworthy that the plant hormone transduction pathway was consistently affected by the neonicotinoid insecticides in the presence and absence of spider mites (Figure 3). 

**Figure 4:**
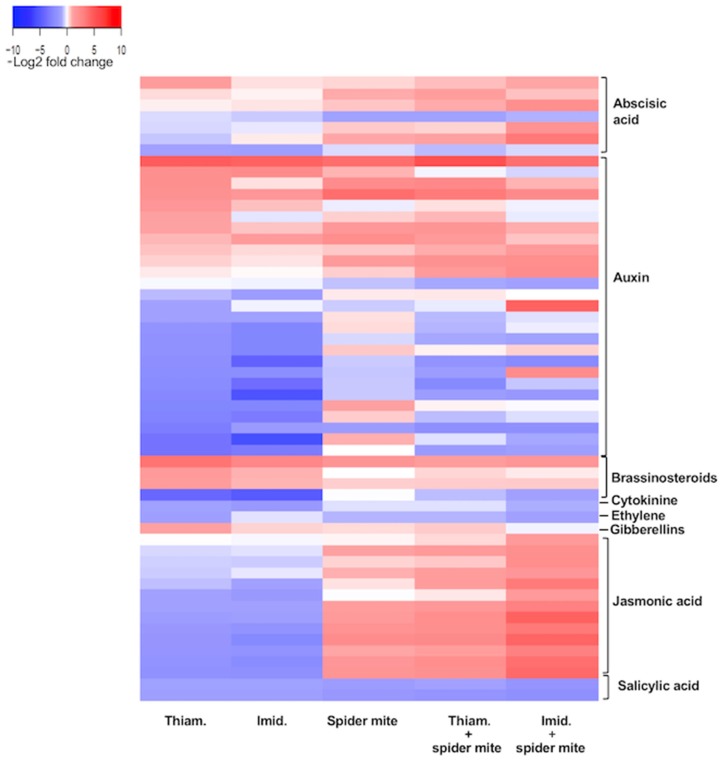
Heat map of DEGs associated with plant hormone transduction pathways in soybean plants exposed to thiamethoxam, imidacloprid, spider mite, and the combination of either neonicotinoid and spider mites. The color key represents log2-transformed fold change; red bars indicate induction of expression, while blue bars represent a decrease in expression.

Genes associated with a number of plant hormones were significantly affected by both insecticides alone and in combination with the herbivore (Figure 4, Supplementary File 2). Most notable of these were JA and SA, which play critical roles in plant–insect and plant–pathogen interactions. Exposure to either insecticide significantly suppressed genes associated with both of these plant hormones. For example, *MYC2* (*Glyma. 09G204500*) transcription factor involved in the JA pathway and JA-mediated resistance to insect pests [40,41,42] was down-regulated following imidacloprid exposure. Spider mite herbivory, in turn, induced genes involved in the JA pathway, which were even further up-regulated in plants exposed to spider mites and treated with imidacloprid. Moreover, all treatments had a suppressive effect on genes involved in the SA pathway. For example, *Glyma.15G062500*, which codes for Pathogenesis-Related Protein 1 (PR-1) involved in plant immune signaling and defense responses against herbivores [43] was down-regulated in soybean plants treated with either insecticide and exposed to spider mites alone or in combination with the neonicotinoids. It is noteworthy that spider mite herbivory suppressed SA-related genes while inducing JA-associated genes, a cross-talk effect between these two hormones common in plant–herbivore interactions [28,29]. Moreover, imidacloprid in combination with spider mite herbivory had the most robust effect on the induction of transcripts associated with the JA pathway. 

Further, auxin, a plant hormone involved in regulation of plant growth [44] as well as plant defense [42,45], appeared to be strongly affected by treatments as well (Figure 4, Supplementary File 2). Both insecticides alone had a similar effect on genes involved in auxin signaling, with roughly half of the differentially expressed transcripts up-regulated and half of them down-regulated. Notably, imidacloprid and thiamethoxam treatments resulted in the suppression of three auxin-responsive protein SAURs (Small Auxin Up RNA), that are involved in several aspects of plant growth and development [46]. These genes, on the other hand, were up-regulated in plants exposed to spider mites alone or in a combination of either insecticide. Moreover, three genes in the brassinosteroid (BR) biosynthesis pathway that is involved in the induction of various detoxifying genes against pesticides [47,48] were strongly up-regulated in thiamethoxam treated plants.

**Figure 5:**
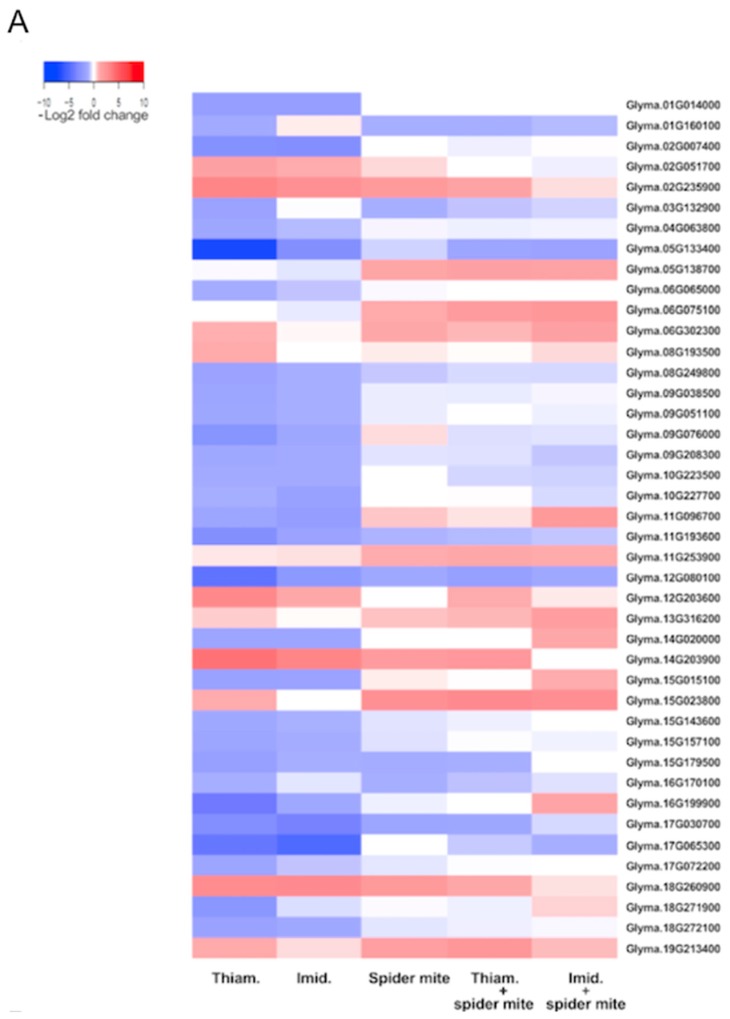

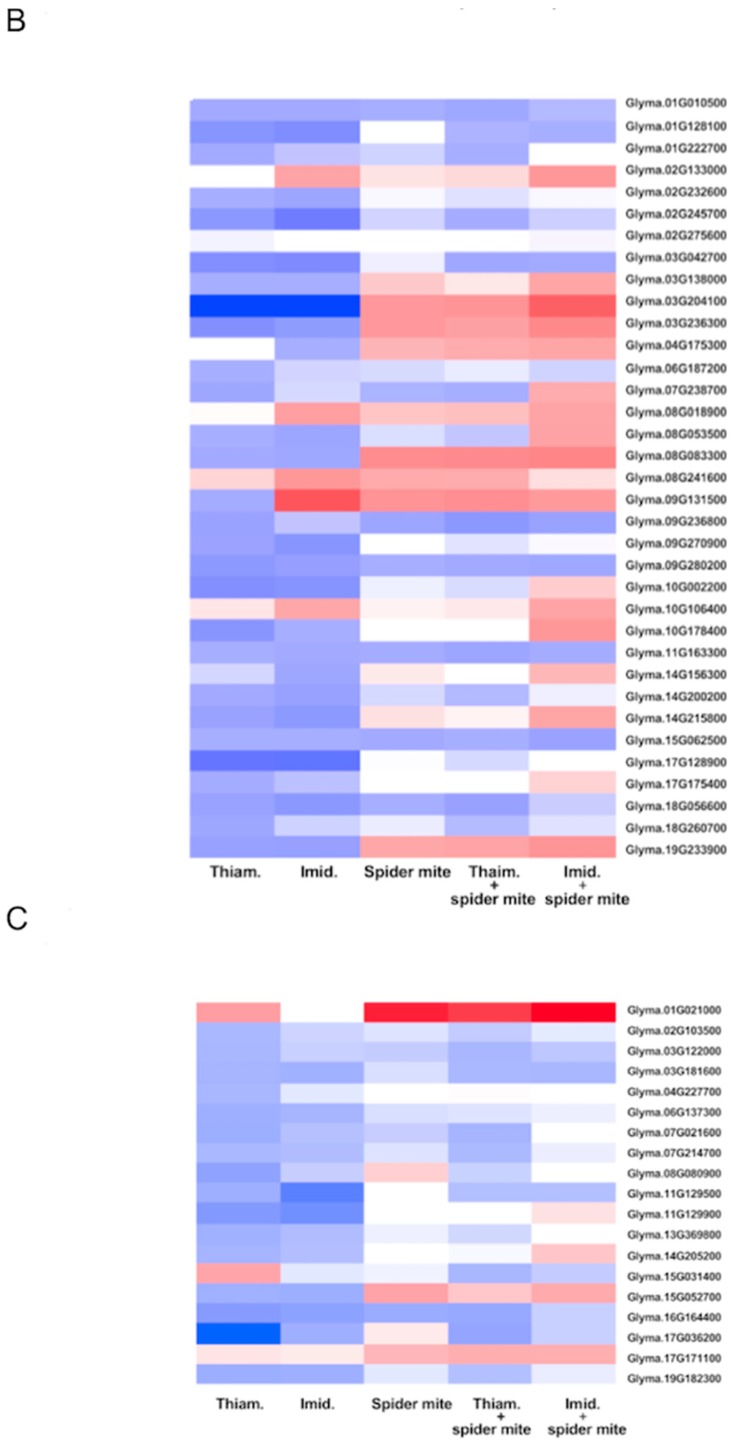
Heat map of DEGs involved in cell wall biosynthesis and metabolism (**A**), plant–pathogen interactions pathway (**B**), and phenylpropanoid biosynthesis pathway (**C**) in soybean exposed to thiamethoxam, imidacloprid, spider mites, and the combination of either neonicotinoid insecticide and spider mites. The color key represents log2-transformed fold change; red bars indicate induction of expression, while blue bars represent a decrease in expression.

Expression patterns of individual genes involved in select pathways and significantly affected by the two neonicotinoids alone and in combination with spider mites were further explored (Figure 5). These comparisons included genes involved in cell wall biosynthesis (Figure 5A), plant–pathogen interactions (Figure 5B) and phenylpropanoid biosynthesis (Figure 5C). A trend common across these plant functions includes a strong downregulation of genes in plants exposed to both neonicotinoids alone. For example, thiamethoxam and imidacloprid suppressed a number of cell wall biosynthesis genes involved in cell wall organization or biogenesis and cellulose metabolic processes (Figure 5A, Supplementary File 3). Spider mite herbivory alone or in combination with either insecticide, on the other hand, had no effect or induced expression of these genes. One notable example of this trend includes a putative *xyloglucan endotransglucosylase/hydrolase* (*XTH*) gene, which cleaves xyloglucan polymers involved in cell wall construction and growth [49]. Expression of this gene was strongly down-regulated by thiamethoxam and imidacloprid, but up-regulated by spider mite herbivory alone or in conjunction with either neonicotinoid insecticide. Further, both thiamethoxam and imidacloprid reduced expression of a number of genes involved in glucose hydrolysis, *GH28, GH17* [50], expression of which was induced in plants exposed to spider mites across neonicotinoid treatments. 

Likewise, both insecticides strongly suppressed genes involved in plant–pathogen interactions (Figure 5B), including a number of genes involved in calcium binding and several *WRKY* transcription factors (TFs) (Supplementary File 4). A number of genes from the Ca^2+^ sensors group encoding calmodulin-like (CMLs) and calmodulin (CAM) proteins that are important in herbivore defense pathways in plants [51,52] were strongly suppressed in response to neonicotinoid treatments. Expression of *Glyma.17G128900*, a putative calcium-binding protein was particularly strongly suppressed by both insecticides but unaffected in plants exposed to the herbivore regardless of insecticides treatments. Further, suppression of multiple genes from the *WRKY* family of TFs that is unique to plants and is involved in regulation of defense responses to pathogens and insects [53,54] was evident in soybean exposed to both insecticides. Several *WRKY* genes including *WRKY19, WRKY33* among others were significantly suppressed by thiamethoxam and imidacloprid, while only *WRKY19* was down-regulated in plants exposed to spider mites. Homologs of these *WRKY* genes were previously implicated in hormone signaling pathways and response to abiotic stresses in Arabidopsis [29,55], and a homolog of *WRKY19*, in particular, has been shown to be a key regulator of cross-talk between SA and JA signaling pathways [56]. 

Expression of genes involved in the phenylpropanoid pathway was also affected by the neonicotinoids and spider mites (Supplementary File 5). The phenylpropanoid pathway is a rich source of metabolites in plants that act as a physical or chemical barrier in plant defense responses toward biotic and abiotic stimuli [57,58]. Thiamethoxam alone suppressed the highest number of transcripts in this pathway, including *caffeic acid 3-O-methyltransferases (COMT), and PAL1* gene that encodes phenylalanine ammonia-lyase and *proxidases*. Imidacloprid treatments suppressed a number of these genes as well, while spider mite herbivory on untreated plants induced expression of only two genes from this pathway, the peroxidase gene and putative *mannitol dehydrogenase*. Effects of both insecticides on the suppression of phenylalanine ammonia-lyase are especially noteworthy as it catalyzes the first step of the phenylpropanoid pathway, which is a key reaction in the control of lignin, flavonoid, and salicylic acid biosynthesis [59]. This gene has also been shown to be affected by neonicotinoid insecticides in previous research [4]. Moreover, several of the other genes associated with the phenylpropanoid pathway that were suppressed by both neonicotinoid insecticides are involved in cross-linkage of lignin precursors [60,61,62]. Mutations in the last steps of lignin biosynthesis through phenylpropanoid pathway including *COMT* can reduce the lignin content, which can affect plant defenses against insects [63,64].

### 2.3. Thiamethoxam Seed Treatments Increase Abundance of Spider Mites

**Figure 6:**
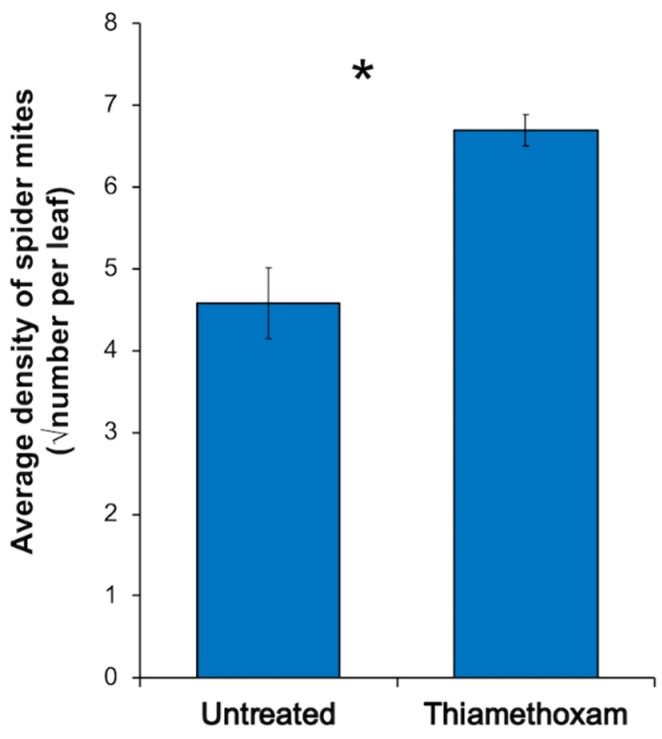
Effect of thiamethoxam seed treatments on density of spider mites on soybean. Values are means ± one standard error. An asterisk designates a significant difference between treatments (*p* < 0.05).

Exposure to thiamethoxam seed treatments significantly increased the density of spider mites on soybean (Figure 6). Spider mites were nearly 50% more abundant on thiamethoxam-treated plants compared to the untreated soybean (*F*_1.38_ = 17.07; *p* < 0.001).

## 3. Discussion

Exposure to either neonicotinoid insecticide in plants free of the herbivore significantly altered transcriptome of soybean plants and suppressed genes regulating key plant functions, such as plant hormone signaling, cell wall macromolecule metabolic process, lignin metabolic process, and defense response. While spider mite herbivory generally restored expression of these transcripts, a prolonged suppression of a suite of genes involved in primary and secondary metabolism is likely to render plants more susceptible to herbivores unaffected by the toxicity of the neonicotinoids. This effect is likely to be especially relevant in seed-treated plants that are exposed to neonicotinoid insecticides from germination and for several weeks before herbivory. A previously published study describing transcriptional responses in soybean exposed to thiamethoxam found similar key pathways including hormone signaling, oxidative stress, and cell wall metabolism to be affected by thiamethoxam, albeit significantly fewer overall changes in soybean transcriptome were reported [5]. 

Thiamethoxam had the greatest impact on soybean transcriptome in our experiment, and the effect of both insecticides on soybean in the absence of spider mites was largely suppressive. Repression of the JA pathway, in particular, is likely to affect subsequent responses of soybean to herbivory and has been shown to play a key role in plant responses to cell content feeding herbivores, such as spider mites [23,27,65]. Both neonicotinoids had a similar and almost exclusively suppressive effect on SA-associated genes as well. Salicylic acid plays an important role in mediating induced plant responses to pathogens in particular [66,67,68] but has also been implicated in plant resistance to herbivores in general [69,70] and spider mites in particular [24]. Interactions between JA and SA are mostly antagonistic, where induction of one of the hormones represses the other [28,71]. Interestingly, while spider mite herbivory resulted in a cross-talk between these two pathways, the combination of imidacloprid exposure and the herbivore had the strongest effect on suppression of two SA-associated genes and simultaneous induction of JA-related transcripts. This was also evident in imidacloprid-treated plants fed upon by spider mites, but the magnitude of this cross-talk was lower. It appears that the combination of the insecticides and spider mite herbivory can have a synergistic effect on the expression of genes in this pathway.

Strong suppression of cell wall biosynthesis and metabolism processes evident in the neonicotinoid-treated plants may explain this synergism. Secondary cell walls and lignin are critical constitutive defensive barriers against pathogens and herbivores, and rapid reinforcement of these structures is an important mechanism for increased plant resistance [34,72,73]. Down-regulation of these genes by neonicotinoid treatments would likely facilitate puncturing cell walls and maneuvering between epithelial cells to access the mesophyll where the mites feed [74,75]. This would also allow cells to be ruptured at a faster rate and enhance the influx of defense-inducing effectors, including JA-associated genes. A similar pattern was identified in grapevine, where increased feeding damage by successful, grapevine-adapted mites induced a strong defensive response, while poor performing, non-adapted mites induced a weak JA response in plants but were unable to survive on the plants [76].

The consequences of cell wall integrity to plant resistance have been explored in previous research. For example, the melon gene VAT promotes resistance to *Aphis gossypii* (Hemiptera: Aphididae) through induction of the hypersensitive response, which increases the lignin and callose content in the cell walls surrounding the aphid stylet path and disrupts aphid feeding [74,77]. Similarly, brown planthoppers, *Nilaparvata lugens* (Hemiptera: Delphacidae), perform better on imidacloprid-treated plants in which lignin and flavonoid synthesis genes were down-regulated by the neonicotinoid treatment [78]. Spider mites are also likely affected by cell wall integrity. In tomato, spider mite feeding up-regulated several cell wall genes, and the repression of secondary cell wall synthesis genes in tomato has been linked to the improved performance of *T. urticae* [27,33,65]. 

It is also noteworthy that genes involved in the biosynthesis of phenylpropanoids and flavonoids, which are highly conserved and are relevant to plants’ induced defenses [65], were suppressed in thiamethoxam-treated soybean. Gene for phenylalanine ammonia-lyase that was suppressed by both insecticides in this study was previously shown to be suppressed in neonicotinoid-treated tomato and cotton exposed to spider mite herbivory, but not in corn [4]. Notably, we found that both insecticides significantly reduced expression of genes involved in phenylpropanoid pathway—peroxidases, and a similar suppression of peroxidases was also reported by a previous study exploring neonicotinoid-induced changes in soybean transcriptome [5]. Peroxidases play a role in oxidative stress response and are also involved in oxidative signal transduction, regulating the redox and Ca^2+^ homeostasis as well as the expression of defense genes [79,80,81]. Reduction in expression of these genes is likely to dampen plant resistance to herbivores and increase the susceptibility of neonicotinoid-treated plants to spider mites.

Effects on soybean transcriptome elicited by neonicotinoid exposure in this study were reflected in reduced resistance of soybean seedlings grown from thiamethoxam-treated seed, which supported higher densities of spider mites than untreated plants. This outcome corroborates earlier reports of neonicotinoid insecticides enhancing spider mite densities in crop plants [4,20]. It is noteworthy that neonicotinoid insecticides exposure does not consistently result in higher spider mite densities, and variability in the propensity of the neonicotinoids to trigger spider mite outbreaks is especially evident in field research. For instance, spider mite abundance on soybean treated with thiamethoxam seed treatments in the field did not differ from untreated plants [82], while cotton exposed to the same insecticide had significantly higher spider mite densities than untreated cotton [4,21]. In yet another study, Ruckert et al. reported outbreaks of Banks grass mite (*Oligonychus pratensis*) on neonicotinoid-treated corn but only when the plants were under stress elicited by water deficit [20]. These examples highlight the complexity of the indirect effects of these insecticides on plant resistance and spider mite fitness and performance. 

We conclude that neonicotinoid insecticides suppress stress-responsive genes, especially those involved in cell wall reinforcement. These results support other observational and experimental evidence that neonicotinoids repress or delay the induction of stress-related genes [4,5]. This dampening of stress gene induction then predisposes plants to increased spider mite herbivory, which heightens the JA mediated defense response to spider mites in treated plants. The strong defense response to mites indicates that the critical determinant of spider mite susceptibility in neonicotinoid-treated plants is likely the repression of constitutive defense genes before herbivory rather than a weakening of the expression of inducible defense genes. This research along with other studies provides evidence that the neonicotinoids alter plants, but there is substantial variability in outcomes of these changes depending on the host plant [3,4], abiotic [5,20], and biotic stresses. Further research is needed to improve our understanding of the complex interactions between these insecticides and plants, and their consequences to plant–herbivore interactions.

## 4. Materials and Methods

### 4.1. Plant Material, Neonicotinoid Treatments, and Spider Mite Colonies

Commercially available soybean *Glycine max* L. (var. S15-L5) seeds were obtained either pretreated only with fungicides (Mefenoxam 0.0113 mg and Fludioxonil 0.0038 mg, Syngenta Crop Protection, LLC, Greensboro, NC, USA) or pretreated with the same fungicides and thiamethoxam (CruiserMaxx^®^ applied at the label rate of 50 g of AI per 100 kg of seed; Syngenta Crop Protection, LLC). All seeds were inoculated with rhizobial bacterial (N-Dure, INTX Microbials, LLC, Kentland, IN, USA), grown in 15-cm pots in Sunshine^®^ soil mix (SunGro, Agawam, MA, USA) within a growth chamber (PGC-10, Percival Scientific Inc., Perry, USA) at a constant temperature of 27 °C, 16 h, 900 µmol/m^2^/s light intensity and 50% humidity. Three weeks after germination, twelve untreated soybean plants at V3 stage (first three trifoliate leaves fully developed) were randomly chosen and exposed to a soil drench using 0.024 g/100mL of water per pot of imidacloprid (Marathon^®^ 75 WP, soluble powder formulation, 750 g of imidacloprid/kg).

Spider mite (*T. cinnabarinus*) colonies were established from naturally occurring greenhouse infestations. Mites were moved onto soybean plants grown from untreated seeds and maintained on soybean plants for at least 3 generations before the onset of experiments.

### 4.2. Impact of Neonicotinoids and Spider Mite Herbivory on Gene Expression

The experiment was a factorial design with three levels of the neonicotinoid insecticide factor (thiamethoxam—Thiam; imidacloprid–Imid; and the untreated control) and two levels of herbivory (spider mites absent; spider mites present). Four weeks after germination, and one week after the imidacloprid treatment, half the soybean plants were exposed to 50 adult spider mites. Mites were moved onto the newest fully expanded trifoliate using a fine-tipped paintbrush, allowed to feed for 24 h, recounted, and then gently wiped from all leaves using a damp KimWipe (Kimberly-Clark). Immediately after removing mites, the entire trifoliate was excised, placed into an individual 15 mL tube (VWR International, Suwanee, GA, USA) and flash frozen in liquid nitrogen. All soybean plants assigned to the absent treatment were sampled within the same hour and in an identical manner. Each of the treatment combinations was replicated three times (N = 18), and each biological replicate was composed of two sub-replicates. All samples were then stored at −80 °C until RNA extractions.

Total RNA was extracted from the entire excised soybean trifoliate by grinding ~100 mg of leaf material in liquid nitrogen with a mortar and pestle. Ground sub-replicates were then combined, and RNA from each biological replicate was extracted using an RNAqeous kit with Plant RNA Isolation Aid (Thermo Fisher Scientific, Waltham, MA, USA) following manufacturers protocols. Total RNA was sent to the Molecular Research LP facility (Shallowater, TX, USA) for quality assessment on a 2100 Bioanalyzer (Aligent, Santa Clara, CA, USA) and for cDNA library synthesis of poly-adenylated mRNA using the Illumina TruSeq RNA Sample Prep V2 LS protocol (Illumina, San Diego, CA, USA). All eighteen samples were sequenced at the facility on one lane of an Illumina HiSeq 2500 v4 machine to produce an average output of 10 million, 150 bp paired-end reads per sample. All raw sequencing reads have been submitted to the NCBI Sequence Read Archive and are available under BioProject ID: PRJNA515005 (available online: http://www.ncbi.nlm.nih.gov/bioproject/515005).

### 4.3. Gene Expression Analysis

Sequence reads were imported into the CLC Genomics Workbench version 11 (Qiagen, Valencia, CA, USA) and mapped to the *G. max* reference genome (Gmax_275_Wm82.a2.v1) [83]. Based on the total read counts for each annotated gene, differential gene expression analyses were conducted using the Empirical Analysis of the DGE tool, which implements the ‘Exact Test’ for two-group comparisons [84]. The transcriptional response of soybean treated plants was compared to non-treated spider mite-free plants (the control) to identify DEGs. Differentially expressed genes were defined as having a fold change ≥2 or ≤−2 with a false discovery rate (FDR) corrected *p*-value < 0.05. 

For functional annotation, GO analysis was performed using AgriGO gene ontology analysis tools [85] to determine overrepresented GO categories in the up- and down-regulated DEGs, and significantly enriched gene ontology GO terms were identified. Pathway analysis was performed using the Kyoto Encyclopedia of Genes and Genomes (KEGG) database [86] to underline the pathways to which the up- and down-regulated DEGs contribute, and pathway enrichment analysis completed using the KEGG Orthology Based Annotation System (KOBAS) server (version v.3) [87]. The overlaps between different sets of DEGs were generated with Bioinformatics & Evolutionary Genomics webtools [20].

### 4.4. Impact of Thiamethoxam on Abundance of Spider Mites

Soybean plants were grown from thiamethoxam-treated treated and untreated seeds as described above. Plants were maintained in mesh insect cages (60 × 60 × 60 cm, Bug Dorm, BioQuip Products, Rancho Dominguez, CA, USA), four plants per cage. All plants were contained within cages from germination until the end of the experiments. Spider mites (*T. cinnabarinus*) used in the experiment were obtained from a colony maintained on soybeans for at least three generations. When plants were at V3 stage (first three trifoliate leaves fully developed, approximately 2 wk following germination) 20 adult females of *T. cinnabarinus* were moved onto three fully expanded leaves of each plant using a fine paintbrush. Spider mites were allowed to colonize and reproduce on the plants for three wk, and plants were destructively sampled at the end of the experiment. Each soybean leaf was excised, and mites were brushed off onto a glass disk using a mite brush (Model 2836M, BioQuip Products, Rancho Dominguez, CA, USA), and counted using a dissecting microscope (Leica EZ4 HD, North Central Instruments, Plymouth, MN, USA). An average density of spider mites was calculated per leaf. Each treatment (thiamethoxam present/absent) was replicated 20 times, and each plant was considered a replicate. Data were transformed (square root) to meet assumptions of normal distribution and homogeneity of variance and analyzed using one-way analysis of variance with treatment as a fixed effect [88].

## Supplementary Materials

Supplementary materials can be found at http://www.mdpi.com/1422-0067/20/3/783/s1.
