# Supplementary material for: Neonicotinoid Insecticides Alter the Transcriptome of Soybean and Decrease Plant Resistance
Source: Int J Mol Sci. 2019 Feb 12;20(3):783. doi: 10.3390/ijms20030783 (PMC6387383; doi:10.3390/ijms20030783)
Supplement: Supplementary file 1 [file ijms-20-00783-s001.zip › Supplementary files/Supplementary File 1.docx]

| **Treatment** | **Biological replication number** | **Total reads (M)** | **Mapped reads (M)** | **Exonic reads (%)** | **Intronic reads (%)** | **Intergenic reads (%)** |
| --- | --- | --- | --- | --- | --- | --- |
| **Control** | 1 | 18.85 | 11.96 | 98.03 | 0.15 | 1.82 |
| **Control** | 2 | 19.34 | 12.11 | 97.91 | 0.20 | 1.89 |
| **Control** | 3 | 24.98 | 16.28 | 97.86 | 0.18 | 1.97 |
| **Mean ± sd** |  | 21.05 ± 3.40 | 13.45 ± 2.45 | 97.93 ± 0.08 | 0.17 ± 0.02 | 1.89 ± 0.07 |
| **Thiamethoxam** | 1 | 19.20 | 12.13 | 98.14 | 0.13 | 1.73 |
| **Thiamethoxam** | 2 | 18.05 | 11.48 | 98.20 | 0.13 | 1.67 |
| **Thiamethoxam** | 3 | 29.68 | 18.97 | 98.16 | 0.13 | 1.72 |
| **Mean ± sd** |  | 22.31 ± 6.40 | 14.19 ± 4.14 | 98.16 ± 0.03 | 0.00 | 1.70 ± 0.03 |
| **Imidacloprid** | 1 | 26.47 | 16.95 | 98.00 | 0.14 | 1.85 |
| **Imidacloprid** | 2 | 28.40 | 17.83 | 98.06 | 0.13 | 1.81 |
| **Imidacloprid** | 3 | 23.48 | 14.82 | 97.94 | 0.15 | 1.91 |
| **Mean ± sd** |  | 26.11 ± 2.47 | 16.53 ± 1.54 | 98.00 ± 0.06 | 0.14 | 1.85 |
| **Spider mite** | 1 | 23.81 | 15.07 | 98.03 | 0.16 | 1.80 |
| **Spider mite** | 2 | 24.12 | 15.22 | 98.02 | 0.14 | 1.84 |
| **Spider mite** | 3 | 17.14 | 10.77 | 98.15 | 0.15 | 1.70 |
| **Mean ± sd** |  | 21.69 ± 3.94 | 13.68 ± 2.52 | 98.06 ± 0.07 | 0.15 ± 0.01 | 1.78 ± 0.07 |
| **Thiamethoxam + spider mite** | 1 | 17.67 | 11.02 | 98.04 | 0.17 | 1.78 |
| **Thiamethoxam + spider mite** | 2 | 12.88 | 8.08 | 98.11 | 0.15 | 1.74 |
| **Thiamethoxam + spider mite** | 3 | 20.91 | 12.97 | 98.10 | 0.19 | 1.71 |
| **Mean ± sd** |  | 17.15 ± 4.03 | 10.69 ± 2.46 | 98.08 ± 0.03 | 0.17 ± 0.02 | 1.74 ± 0.03 |
| **Imidacloprid + spider mite** | 1 | 21.80 | 13.79 | 97.92 | 0.14 | 1.93 |
| **Imidacloprid + spider mite** | 2 | 28.10 | 17.69 | 97.99 | 0.15 | 1.87 |
| **Imidacloprid + spider mite** | 3 | 23.97 | 15.25 | 97.89 | 0.17 | 1.94 |
| **Mean ± sd** |  | 24.62 ± 3.2 | 15.57 ± 1.97 | 97.93 ± 0.05 | 0.15 ± 0.01 | 1.91 ± 0.03 |

## **Supplementary File 1: Summary of RNA-seq reads from control soybean plants and plants in response to thiamethoxam, imidacloprid, spider mite and their interactions mapped to the soybean genome.** Unique RNA-seq, reads mapping to exons, introns, and intergenic regions are shown as the percentage of total reads distributed to these annotated regions of the soybean genome (Gmax_275_Wm82.a2.v1).
